# Antidiabetic Agent DPP-4i Facilitates Murine Breast Cancer Metastasis by Oncogenic ROS-NRF2-HO-1 Axis *via* a Positive NRF2-HO-1 Feedback Loop

**DOI:** 10.3389/fonc.2021.679816

**Published:** 2021-05-26

**Authors:** Rui Li, Xin Zeng, Meihua Yang, Xiaohui Xu, Jinmei Feng, Liming Bao, Bingqian Xue, Xin Wang, Yi Huang

**Affiliations:** ^1^ Chongqing Key Laboratory of Child Infection and Immunity, Ministry of Education Key Laboratory of Child Development and Disorders, National Clinical Research Center for Child Health and Disorders, China International Science and Technology Cooperation Base of Child Development and Critical Disorders, Children’s Hospital of Chongqing Medical University, Chongqing, China; ^2^ Department of Neurosurgery, Xinqiao Hospital of Third Military Medical University, Chongqing, China; ^3^ Department of Pathology, School of Medicine, University of Colorado Anschutz Medical Campus, Aurora, CO, United States; ^4^ Department of Laboratory Medicine, The People’s Hospital of Guangxi Zhuang Autonomous Region, Nanning, China

**Keywords:** DPP-4 inhibitor, NRF2, HO-1 (heme oxygenase-1), metastasis, breast cancer

## Abstract

Cancer has been as one of common comorbidities of diabetes. Long-term antidiabetic treatment may potentially exert uncertain impacts on diabetic patients with cancer including breast cancer (BC). Dipeptidyl peptidase-4 inhibitors (DPP-4i) are currently recommended by the AACE as first-line hypoglycemic drugs in type 2 diabetes mellitus (T2DM). Although the safety of DPP-4i has been widely evaluated, the potential side-effects of DPP-4i in cancer metastasis were also reported and remain controversial. Here, we revealed that Saxagliptin (Sax) and Sitagliptin (Sit), two common DPP-4i compounds, potentially promoted murine BC 4T1 metastasis *in vitro* and *in vivo* under immune-deficient status. Mechanically, we observed that DPP-4i treatment induced aberrant oxidative stress by triggering ROS overproduction, as well as ROS-dependent NRF2 and HO-1 activations in BC cells, while specific inhibition of ROS, NRF2 or HO-1 activations abrogated DPP-4i-driven BC metastasis and metastasis-associated gene expression *in vitro*. Furthermore, ALA, a NRF2 activator significantly promoted BC metastasis *in vitro* and *in vivo*, which can be abrogated by specific HO-1 inhibition *in vitro*. Moreover, specific HO-1 inhibition not only reversed DPP-4i-induced NRF2 activation but also abrogated ALA-induced NRF2 activation, resulting in a decrease of metastasis-associated genes, indicating a positive-feedback NRF2-HO-1 loop. Our findings suggest that DPP-4i accelerates murine BC metastasis through an oncogenic ROS-NRF2-HO-1 axis *via* a positive-feedback NRF2-HO-1 loop. Therefore, this study not only offers novel insights into an oncogenic role of DPP-4i in BC progression but also provides new strategies to alleviate the dark side of DPP-4i by targeting HO-1.

## Introduction

Accumulating evidences suggest that diabetes increases incidence of human cancers including breast cancer (BC) (1). Long-term treatment of antidiabetic drugs may potentially have uncertain impacts on comorbid BC in diabetic patients. Therefore, improved understanding of the effects of antidiabetic agents in BC cells should open new avenues for minimizing the risk of antidiabetic agents in diabetic patients with BC (2). Dipeptidyl peptidase-4 inhibitors (DPP-4i), such as saxagliptin (Sax) and sitagliptin (Sit), are currently recommended by the American Association of Clinical Endocrinologists (AACE) as first-line hypoglycemic treatment in type 2 diabetes mellitus (T2DM). Although the safety of DPP-4i has been widely evaluated, the potential side effects of DPP-4i in cancer metastasis were also reported and remains controversial (3, 4). A previous report suggested a potential anti-tumor role of DPP-4 inhibition in improvement of tumor immunity by regulating CXCL10-mediated lymphocyte trafficking in mice (5), whereas our recent finding revealed an oncogenic role of DPP-4i in human cancers including BC through NRF2-mediated anti-oxidative stress (6). Thus, the potential side-effects of antidiabetic agents DPP-4i in BC metastasis should be further clarified.

The cancer immunoediting concept has provided a critical insight into the crosstalk between tumor cells and immune system during the cancer development (7–11). Although there is no doubt that immunity is critical to tumor progression, tumor cells as one of the major components of tumor microenvironment, was shown to exert major contribution to tumor progression by remodeling the tumor microenvironment (7–11). Thus, better understanding the effect of antidiabetic DPP-4i on existing tumors and the underlying mechanism not only would offer novel insights into its potential role in tumor progression but also may provide new strategies to alleviate the dark side of DPP-4i in diabetic patients with cancer.

Here, we utilized a murine BC cell line 4T1, that is well known to mimic the metastatic and advanced stages of human BC (11), to investigate the effect of Sax and Sit on BC metastasis *in vitro* and *in vivo*. Then, we further investigated the possible mechanism underlying how DPP-4i regulates BC metastasis.

## Materials and Methods

### Cell Lines, Cell Culture, and Reagents

Murine BC cell line 4T1 was obtained from the American Tissue Culture Collection (ATCC) as our previous reports (11). Cells were cultured in RPMI 1640 (Gibco BRL, Rockville, MD, USA) supplemented with 10% fetal bovine serum, 100 U/ml penicillin, and 100 μg/ml streptomycin at 37°C in a humidified chamber containing 5% CO_2_. Neh2-Luciferase reporter vectors for NRF2/ARE activation were reserved by our lab as described previously (12). Saxagliptin (Sax), Sitagliptin (Sit), and Heme oxygenase 1 (HO-1) specific inhibitor HO-1-IN-1 hydrochloride (10 mM in DMSO) were purchased from MedChemExpress (MCE) (Houston, TX, USA). ROS scavenger N-acetylcysteine (NAC) and NRF2 specific inhibitor ML-385 were purchased from AbMole Bioscience (Houston, TX, USA). Alpha-lipoic acid (ALA, a NRF2 activator) was commercially obtained from Dandong Yichuang Co, China as previous describes (12). All chemical reagents were purchased from Sigma-Aldrich (St Louis, MO, USA) unless otherwise indicated.

### Cell Migration and Cell Invasion Assays

Cell migration and cell invasion assays were performed in 24-well non-coated or Matrigel-coated Transwell chambers (8-µm pore size, Corning, NY, USA) as described previously (12–14). Briefly, 4.5 × 10^4^ cells for cell migration or 1 × 10^5^ cells for cell invasion were plated in the upper chamber with 200 μl of serum-free medium, and 800 μl medium supplemented with 10% FBS was used as a chemoattractant in the bottom chamber. 4T1 cells were treated with Sax (0, 0.2, 0.4 μM), Sit (0, 0.6, 1.2 μM) or ALA (0, 40, 60 μM) for 24 hours, and then were fixed and stained with Crystal Violet Staining Solution (Beyotime, Haimen, China). For pharmacological intervention assays, 4T1 cells were co-treated with Sax (0.4 μM) or Sit (1.2 μM), and NAC (0, 2.5, 5 mM), or ML-385 (0, 5, 10 µM), or HO-1 inhibitor (0, 5,10 µM), respectively. For HO-1 blockage assay, cells were co-treated with ALA (60 μM) and HO-1 inhibitor (0, 5, 10 µM), and cell migration and cell migration assays were performed as above mentioned. The images of the migrated or invaded cells were captured and cell number was counted in 5 to 10 random fields for each group and summarized as mean ± standard deviation (SD) for statistical analysis.

### Spontaneous Breast Cancer Metastasis Mouse Model

NOD-SCID mice (6–8 weeks, Female, SPF degree, 22 ± 3 g) were purchased from Beijing HFK Bioscience Co (Beijing, China). All mice were housed and maintained under specific pathogen-free (SPF) conditions as our described previously (12, 13). All procedures were approved by the Institutional Animal Care and Use Committee of Children’s Hospital of of Chongqing Medical University. A spontaneous BC metastasis mouse model was established as previously described (11). Briefly, 4T1 cells (1 × 10^5^) in 100 µl PBS buffer were subcutaneously injected into in the left mammary fat pad of NOD-SCID mice. After 3 to 5 days, 4T1-bearing mice were randomly divided into two groups to receive 0.9% NaCl or 15 mg/kg Sax orally daily (n = 3–5 mice/group). For ALA intervention *in vivo*, 4T1-bearing mice were randomly divided into two groups to receive intraperitoneal (i.p.) administration of 0.9% NaCl or ALA (80 mg/kg in 0.9% NaCl) three times per week (n = 3–5 mice/group). At the end of experiments, experimental mice were sacrificed, and liver and lung tissues were harvested for the analysis of H&E and immunohistochemistry staining.

### Reactive Oxygen Species (ROS) Measurement

Intracellular ROS and mitochondrial ROS (mROS) were measured by flow cytometry according to procedures as described previously (6, 12). Indicated cells were treated with 10 μM DHE (Dihydroethidium) (Sigma-Aldrich) for 30 min at 37°C. After washing with PBS, cells were resuspended in ice-cold PBS for flow cytometry analysis. mROS was measured using 5.0 μM MitoSoX Red probe (Thermo Fisher Scientific) according to the manufacturer’s instructions. All stained cells were analyzed on a FACS Calibur flow cytometer (BD Bioscience) and data analyzed with FlowJo software (Tree Star, Ashland, OR).

### Intracellular Adenosine Triphosphate Level Assay

Intracellular a**denosine** t**riphosphate (**ATP) levels were measured by ATP Assay Kit (Beyotime, Haimen, China) in accordance with the manufacturer’s instructions as described previously (6, 12). Briefly, cells in a six-well were treated with or without Sax or Sit for 24 h and homogenized with ice-cold lysis buffer. After centrifuged for 5 min at 12,000*g*, 4°C, the supernatant was used for RLU value detection using a Synergy H1 microplate reader (Bio Tek).

### Intracellular Reduced Glutathione/Oxidized Glutathione ratio and NADP+/NADPH Ratio Analysis

The NADP+/NADPH ratio and reduced glutathione (GSH)/oxidized glutathione (GSSG) ratio were determined using the NADP+/NADPH Assay Kit (Beyotime, Haimen, China) and GSH/GSSG Ratio Detection Assay Kit (Beyotime, Haimen, China), respectively. These assays were performed to examine the oxidative status of the cells pretreated with or without Sax or Sit according to the manufacturer’s instructions as described previously (6, 12).

### RNA Isolation and Quantitative Real-Time PCR (qRT-PCR)

RNA isolation and qRT-PCR were performed as described previously (12–17). Total RNA from harvested cells was isolated using Tripure Isolation Reagent (Roche, Mannheim, Germany). 0.5-1.0 µg total RNA was reverse-transcribed into cDNA using the PrimeScript™ RT reagent Kit with gDNA Erase r(Takara, Japan) according to the manufacturer’s instructions. qRT-PCR was performed with QuantiNova SYBR Green PCR Kit (Qiagen, Germany) on CFX Connect™ Real-Time System (BIO-RAD) according to the manufacturer’s instructions. The relative gene expressions were normalized to the house-keeping β-actin gene and calculated using the 2^−ΔΔCt^ method. The details of the primers are listed in **Supplementary Table S1**.

### Western Blotting

4T1 cells were treated with indicated regents and then subject to Western blotting analysis using RIPA buffer (Beyotime, Haimen, China) as described previously (12–16). Protein lysates after SDS-PAGE were blotted onto PVDF membranes, blocked in QuickBlock™ Blocking Buffer for Western Blot (Beyotime, Haimen, China) and followed by primary antibody incubation. Blots were washed with TBST and detected with the ECL system. All antibodies used in this study are listed in **Supplementary Table S2**.

### Luciferase Reporter Assays

Luciferase reporter assay was performed as our described previously (12, 13). Briefly, cells were seeded in 96-well plates at approximately 1 × 10^4^ cells per well and then transfected with the Neh2-Luciferase reporter vectors (90 ng) using X-tremeGENE HP DNA Transfection Reagent (Roche, Germany). pRL-TK *Reniila* plasmids (10 ng) (Promega, Madison, WI, USA) were co-transfected to normalize transfection efficiency. After transfection 16 to 18 h, indicated regents, such as inhibitors or activators, were added as stimulation groups. After another 24 h incubation, the Firefly and Renilla luciferase activities were quantified using the Dual-Glo^®^ Luciferase Assay System (Promega, Madison, USA). The relative luciferase (Luc) activity was present as the fold-change of in Firefly luciferase activity after normalization to the Renilla luciferase activity.

### H&E Staining and Immunohistochemistry (IHC)

H&E and IHC staining were performed as described previously (6, 12–16). Briefly, lung or liver tissues were fixed with 10% buffered formalin and embedded in paraffin. Tissue sections (4 μm) were subjected to H&E staining. IHC staining was performed using Elivision plus Polyer HRP IHC Kit (Maixin, Fujian, China) and DAB kit (ZSGB-Bio, Beijing, China) according to the manufacturer’s instructions.

### Immunofluorescence

**Immunofluorescence (**IF) was performed as described previously (6, 12, 13). Briefly, cells were fixed with 4% paraformaldehyde for 30 min and blocked with QuickBlock™ Blocking Buffer (Beyotime, Haimen, China) for 15 min at room temperature. Then cells were incubated with primary antibodies at 4°C overnight, followed by incubation for 2 h at room temperature with AF555 or AF647-conjugated secondary antibody (Biolegend). Nuclei were counterstained with 4′,6-diamidino-2-phenylindole (DAPI). Images were captured using a Nikon AIR Confocal Laser Microscope (Nikon, Minato, Japan) and mean fluorescent intensity (MFI) was measured by a NIS elements AR analysis software version 5.01. All antibodies used in this study are listed in **Supplementary Table S2**.

### Statistics

Statistical analysis was carried out with the GraphPad Prism 7.0 (GraphPad Software) as previously described (12, 13). All data were expressed as means ± SD. The significance of difference between groups was determined by unpaired two-tailed Student’s *t* test or the one-way analysis of variance (ANOVA). The value of p < 0.05 was considered statistically significant.

## Results

### DPP-4i (Sax and Sit) Facilitates 4T1 BC Cells Metastasis *In Vitro* and *In Vivo*

**Figure 1:**
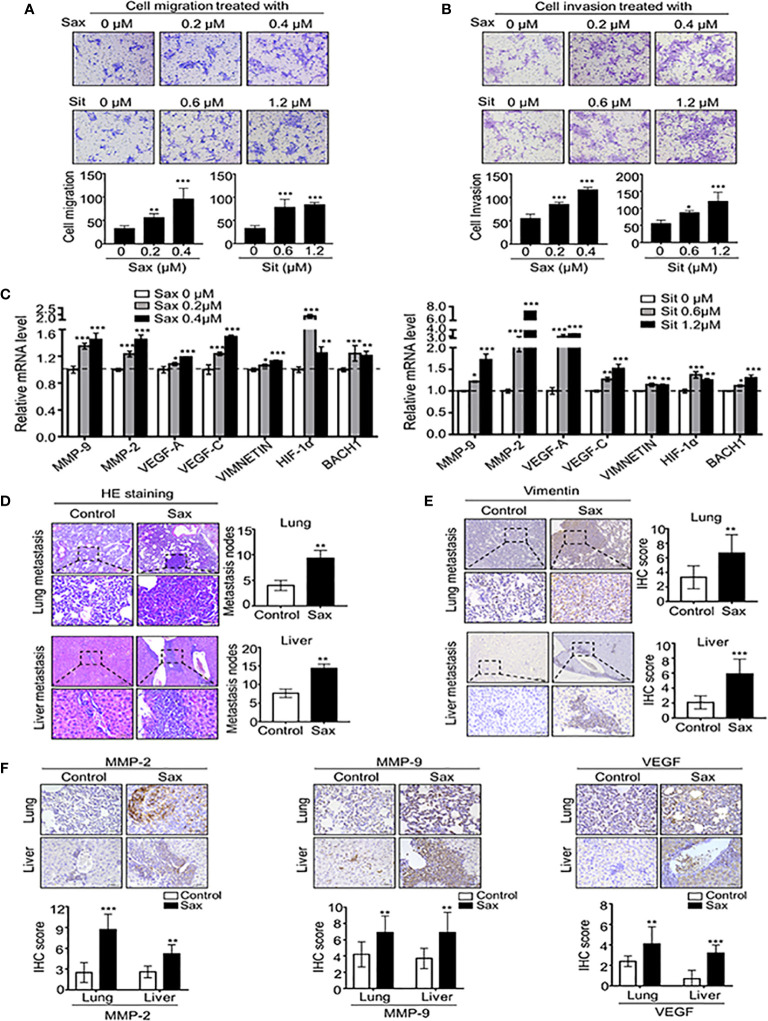
DPP-4i(Sax and Sit)promotes murine breast cancer metastasis *in vitro* and *in vivo*. **(A, B)** DPP-4i (Sax and Sit) facilitates cells migration and invasion *in vitro*. 4T1 cells were subject to cell migration assay **(A)** and cell invasion analysis **(B)** upon treatment of Saxagliptin (Sax) or Sitagliptin (Sit). Migration or invasion cells were counted in 5 to 10 random fields. **(C)** DPP-4i increases metastasis-associated gene levels. 4T1 cells were co-treated with or without DPP-4i and metastasis-associated gene levels were detected by qRT-PCR. β-actin gene was as an internal control. **(D)** DPP-4i accelerates BC lung and liver metastasis *in vivo*. 1.0 × 105 4T1 cells in 100 μl PBS buffer were injected into in the left mammary fat pad of female NOD-SCID mice. Post-injection 3 to 5 days, mice were randomly allocated to the indicated groups (n = 3–5 mice/group) and treated with 0.9% NaCl (control) or 15 mg/kg Sax via oral gavage daily. After the indicated time, lungs and livers were collected, and metastatic nodes were counted by H&E staining **(D)**. Micro-metastasis marker vimentin was detected by IHC staining **(E)**. DPP-4i enhances metastasis-associated gene expression in vivo **(F)**. 4T1-bearing mice were treated with DPP-4i (Sax) as above mentioned and metastasis-associated proteins MMP-9, MMP-2 and VEGF were detected by IHC staining in lung and liver metastatic tissues. Data are presented as mean ± SD of three independent experiments. Representative images are shown. Scale bar: 50 μm. *p < 0.05, **p < 0.01, and ***p < 0.001 between the indicated groups determined by unpaired Student’s t test or the one-way analysis of variance (ANOVA).

To understand the potential role of DPP-4i in BC progression, we investigated the effect of two DPP-4i compounds, Sax and Sit on BC metastasis using a murine BC cell line 4T1 cells, which was used to mimic the metastatic and advanced stages of human BC (11). Firstly, we observed that Sax and Sit markedly enhanced the abilities of BC cell migration and cell invasion *in vitro* (**Figures 1A, B**). Furthermore, we observed that DPP-4i treatment significantly enhanced a serial of metastasis-associated gene levels including HIF-1α, MMP-2, MMP-9 VEGF-A, VEGF-C, VIMENTIN, and BACH-1 (**Figure 1C**). These results indicate that DPP-4i promotes BC migration and invasion *in vitro*.

To further demonstrate the direct role of DPP-4i on tumor metastasis *in vivo*, we subcutaneously injected 4T1 cells into the left mammary fat pad of severe combined immunodeficient NOD-SCID mice to establish a spontaneous metastasis mice model. We observed that DPP-4i treatment significantly enhanced lung and liver metastasis of BC cells *in vivo* (**Figure 1D**). Moreover, vimentin, a micro-metastasis marker and metastasis-associated genes MMP-2, MMP-9, and VEGF levels were markedly increased in lung and liver micro-metastasis nodes (**Figures 1E, F**). Thus, these data indicate that DPP-4i increases BC migration and invasion by increasing metastasis-associated gene expression, thereby facilitating metastasis *in vivo*.

### ROS-Induced Aberrant Oxidative Stress Contributes to DPP-4i–Driven BC Metastasis

**Figure 2:**
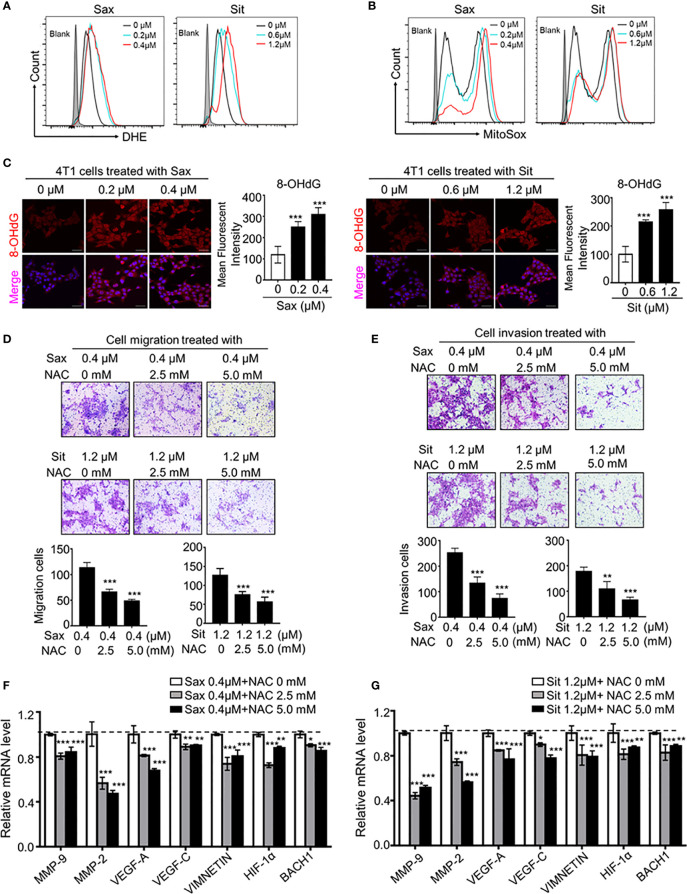
ROS scavenger reverses DPP-4i-driven metastasis. **(A, B)** DPP4i triggers ROS overproduction. 4T1 cells were treated with DPP-4i. Intracellular ROS and mROS were detected in DPP-4i-treated BC cells by FCM using DHE staining **(A)** and MitoSox staining **(B)**. **(C)** DPP-4i induces DNA damage in BC cells. 4T1 cells were treated with DPP-4i and DNA damage marker 8-oxo-deoxyguanosine (8-OHdG) was detected by IF staining. **(D, E)** ROS scavenger abrogates DPP-4i -driven cell migration and invasion *in vitro*. 4T1 cells were subject to cell migration **(D)** and cell invasion **(E)** assays upon co-treatment of DPP-4i and NAC for 24 h. Migration or invasion cells were counted in 5 to 10 random fields. **(F, G)** ROS scavenger abrogates DPP-4i-driven metastasis-associated gene levels. 4T1 cells were co-treated with or without DPP-4i and NAC for 4 h and metastasis-associated gene levels were detected by qRT-PCR. β-actin gene was as an internal control. Data are presented as mean ± SD of three independent experiments. Representative images are shown. Scale bars: 50 μm. *p<0.05, **p<0.01, and ***p<0.001 between the indicated groups determined by one-way analysis of variance (ANOVA).

Given the role of DPP-4i in the regulation to oxidative stress, we sought to know whether oxidative stress is involved in DPP-4i-induced BC metastasis. Firstly, we observed that intracellular ROS and mROS productions were significantly enhanced in DPP-4i-treated BC cells (**Figures 2A, B**), indicating that DPP-4i induces the robust release of ROS in BC cells. Then, we also assessed the effect of DPP-4i on oxidative stress status. We observed that DPP-4i significantly impaired ATP production and decreased NADP+/NADPH ratio, but markedly increased the GSH/GSSG ratio in BC cells (**Supplemental Figure S1**). Meanwhile, DNA damage marker 8-OHdG levels were markedly enhanced after DPP-4i treatment (**Figure 2C**). These data suggest that DPP-4i triggers aberrant oxidative stress by inducing ROS over production in BC cells.

To demonstrate the essential role of ROS in DPP-4i-induced BC metastasis, we applied the ROS scavenger-NAC intervention to assess the effect of ROS inhibition on DPP-4i–driven BC metastasis *in vitro*. As shown in **Figures 2D, E**, NAC treatment significantly abrogated DPP-4i–driven BC cell migration and invasion with a dose-dependent manner. Furthermore, DPP-4i–driven metastasis-associated gene levels were also inhibited by NAC treatment (**Figures 2F, G**), indicating that ROS plays an oncogenic role in DPP-4i-induced BC metastasis. Therefore, these results suggest that DPP-4i drives BC metastasis by triggering oxidative stress *via* ROS overproduction.

### DPP-4i Induces Aberrant NRF2 Activation by a ROS-Dependent Manner

**Figure 3:**
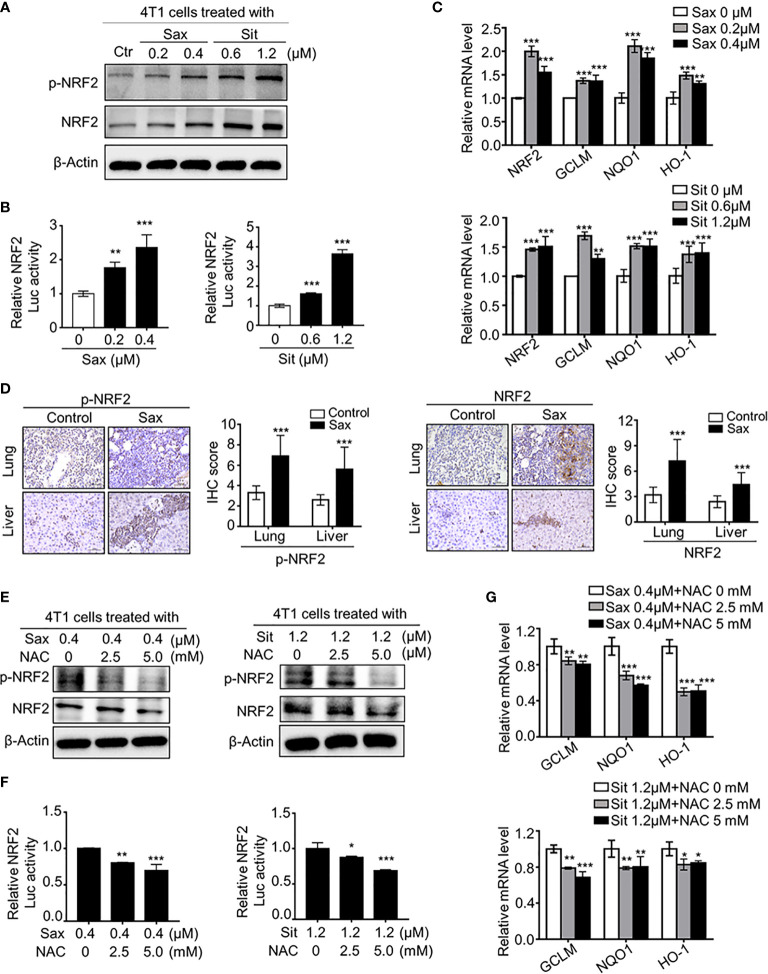
DPP-4i induces ROS-dependent NRF2 activation. **(A–C)** DPP-4i enhances aberrant NRF2 activation of 4T1 cells in vitro. 4T1 cells were treated with DPP-4i. NRF2 and p-NRF2 expressions were detected by Western blotting **(A)**. NRF2/ARE transcriptional activation was analyzed by luciferase reporter gene assay **(B)** and NRF2-responsive genes levels were analyzed by Real-time PCR **(C)**. β-actin gene was as an internal control. **(D)** DPP-4i induces aberrant NRF2 activation in vivo. p-NRF2 and NRF2 proteins were detected by IHC staining in metastatic lung and liver tissues. **(E–G)** ROS scavenger abrogates DPP-4i-driven NRF2 activation. 4T1 cells were co-treated with DPP-4i and NAC. NRF2 and p-NRF2 expressions were detected by western blotting **(E)**. NRF2/ARE transcriptional activation was analyzed by luciferase reporter gene assay **(F)** and NRF2-responsive genes levels were analyzed by real-time PCR **(G)**. β-actin gene was as an internal control. Representative images are shown. Scale bars: 50 μm. Data are presented as mean ± SD of three independent experiments. *p < 0.05, **p < 0.01, and ***p < 0.001 between the indicated groups determined by unpaired Student’s *t* test or the one-way analysis of variance (ANOVA).

It has been shown that DPP-4i reduced ROS-mediated oxidative stress by NRF2 activation in some human cancer cells (6), we further investigated the correlation between ROS and NRF2 in DPP-4i-treated BC cells. We found that DPP-4i significantly increased NRF2 and p-NRF2 expression (**Figure 3A**) and ARE-driven NRF2 transcriptional activation (**Figure 3B**). Meanwhile, NRF2-responsive genes expressions were significantly upregulated upon DPP-4i treatment (**Figure 3C**). Then, we further observed that NRF2 and p-NRF2 expressions were also increased in lung and liver metastasis tissues after DPP-4i treatment (**Figure 3D**). These data suggest that DPP-4i triggers aberrant NRF2 activation.

To further clarify the relationship between ROS and NRF2 activation, we investigated whether ROS inhibition could reverse DPP-4i-driven NRF2 activation. By ROS scavenger NAC intervention, we found that NAC treatment significantly attenuated NRF2 and p-NRF2 levels (**Figure 3E**), as well as NRF2/ARE luciferase activation (**Figure 3F**) in DPP-4i–treated BC cells. Furthermore, NRF2-responsive gene levels were significantly attenuated after NAC treatment in DPP-4i–treated BC cells (**Figure 3G**), indicating that DPP-4i induces NRF2 activation by triggering ROS. Collectively, these data indicate that DPP-4i induces aberrant NRF2 activation *via* ROS-dependent manner.

### Oncogenic NRF2 Activation in DPP-4i-Driven BC Metastasis

**Figure 4:**
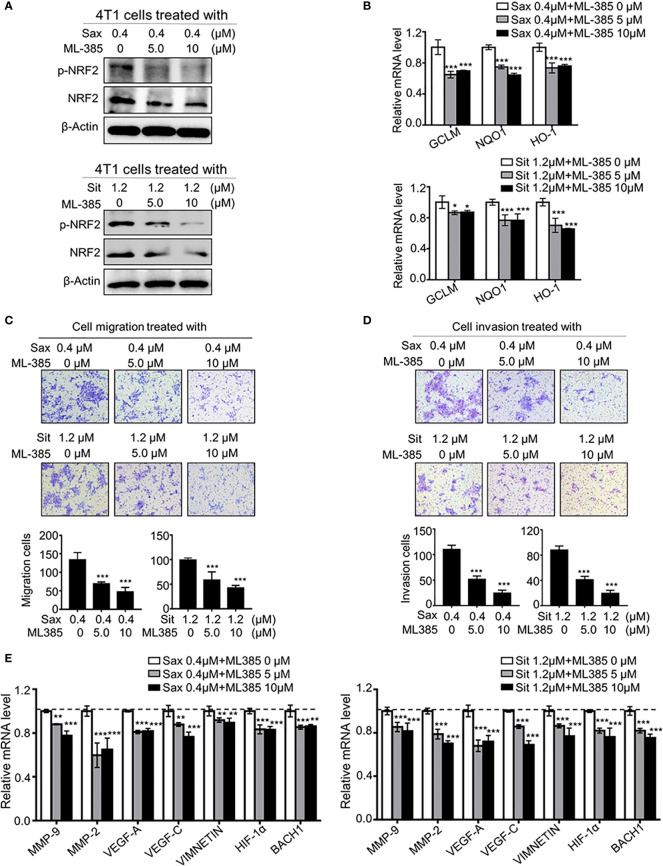
Blockage NRF2 reverses DPP-4i-driven metastasis *in vitro* **(A, B)** NRF2 inhibition reverses DPP-4i-driven NRF2 activation. 4T1 cells were co-treated with DPP-4i and NRF2 specific inhibitor ML-385 for 4h. NRF2 and p-NRF2 expressions were detected by Western blotting **(A)** and NRF2-responsive genes levels were detected by qRT-PCR **(B)**. β-actin gene was as an internal control. **(C, D)** NRF2 blockage attenuates DPP-4i–driven cell migration and invasion *in vitro*. 4T1 cells were subject to cell migration **(C)** and cell invasion **(D)** upon co-treatment with DPP-4i and ML-385 for 24 h. Migration or invasion cells were counted in 5 to 10 random fields. **(E)** NRF2 blockage abrogates DPP-4i-driven metastasis-associated gene levels. 4T1 cells were co-treated with or without DPP-4i and ML-385 and metastasis-associated gene levels were detected by qRT-PCR. β-actin gene was as an internal control. Data are presented as mean ± SD of three independent experiments. Scale bar: 50 μm. Representative images are shown. *p < 0.05, **p < 0.01, and ***p < 0.001 between the indicated groups determined by one-way analysis of variance (ANOVA).

To define the role of NRF2 in DPP-4i-driven BC metastasis, we used ML-385, a specific NRF2 inhibitor, to explore whether pharmaceutical NRF2 inhibition could reverse DPP-4i-driven BC metastasis. We found that ML-385 treatment significantly attenuated NRF2, p-NRF2 expression (**Figure 4A**) and NRF2-responsive gene levels (**Figure 4B**) in DPP-4i-treated BC cells. Notably, DPP-4i-driven BC cell migration and invasion were significantly abrogated by ML-385 treatment with a dose-dependent manner (**Figures 4C, D**). Furthermore, DPP-4i–driven metastasis-associated gene levels were significantly attenuated after ML-385 treatment in BC cells (**Figure 4E**). These data indicate that NRF2 inhibition reverses DPP-4i–driven BC metastases *in vitro.*

**Figure 5:**
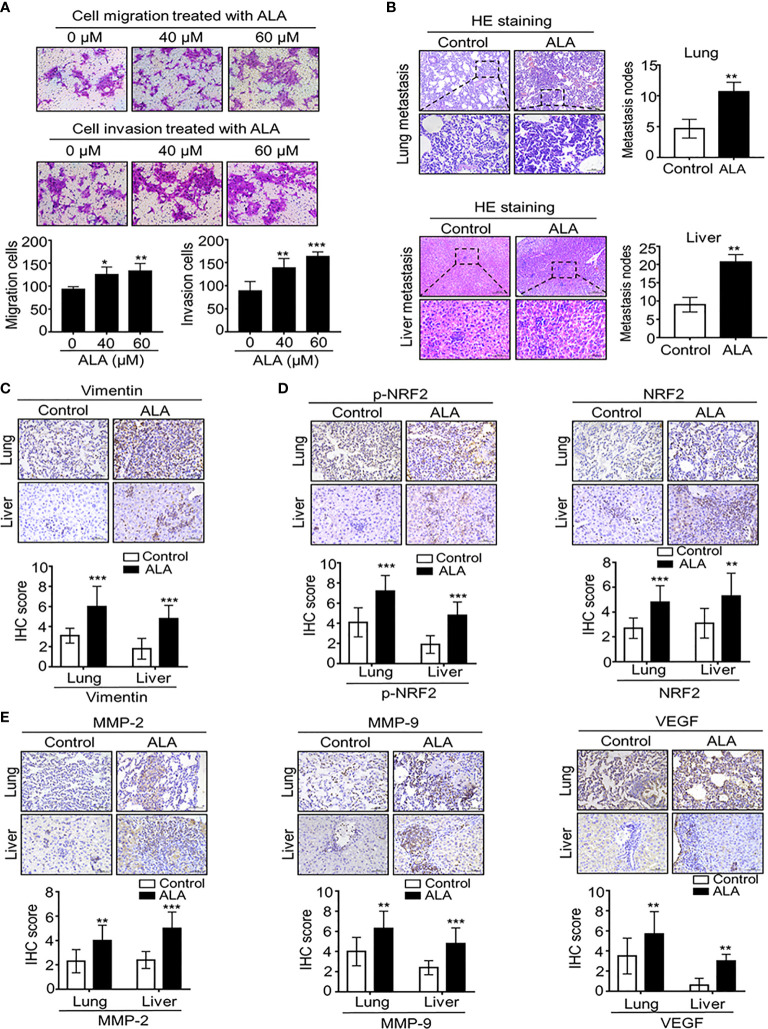
Pharmaceutical NRF2 activation promotes BC metastasis in vitro and in vitro. **(A)** NRF2 activator ALA promotes BC cell migration and cell invasion. 4T1 cells were subject to cell migration and cell invasion assay upon ALA treatment. Migration or invasion cells were counted in 5 to 10 random fields. **(B, C)** ALA accelerates BC lung and liver metastasis *in vivo*. 1.0 × 10^5^ 4T1 cells in 100 μl PBS buffer were injected into in the left mammary fat pad of female NOD-SCID mice. Post-injection 3 to 5 days, experimental mice were peritoneally injected (i.p.) with or without ALA (80mg/kg) three times per week as described in M&M. After the indicated time point, lungs and livers were collected and metastatic nodes were counted by H&E staining **(B)**. Micro-metastasis marker vimentin was detected by IHC staining **(C)**. **(D)** ALA induces NRF2 activation in vivo. 4T1-bearing mice were treated with ALA as above mentioned and p-NRF2 and NRF2 were detected by IHC staining in metastatic lung and liver tissues. **(E)** ALA enhances metastasis-associated gene expression *in vivo*. 4T1-bearing mice were treated with ALA as above mentioned and metastasis-associated proteins MMP-9, MMP-2 and VEGF were detected by IHC staining in lung and liver metastatic nodes. Data are presented as mean ± SD of three independent experiments. Scale bar: 50 μm. Representative images are shown. *p < 0.05, **p < 0.01 and ***p < 0.001 between the indicated groups determined by unpaired Student’s *t*-test or the one-way analysis of variance (ANOVA).

To further understand the exact role of NRF2 activation in BC metastasis, we investigated the direct effect of NRF2 activator on BC cell migration and invasion. Using α-lipoic acid (ALA), a well-characterized NRF2 activator, we found that pharmacological ALA treatment significantly promoted cell migration and cell invasion with a dose-dependent manner in BC cells (**Figure 5A**). Meanwhile, NRF2-responsive gene levels were also significantly increased after ALA treatment (**Supplementary Figure S2A**), indicating that pharmacological NRF2 activation promotes cell migration and invasion *in vitro*.

In complementary *in vivo* metastatic model, ALA was intraperitoneally injected into 4T1-bearing mice to demonstrate the direct effect of NRF2 activation on BC metastasis. We found that ALA treatment significantly enhanced BC lung and liver metastasis (**Figure 5B**) while micro-metastasis marker vimentin was markedly increased in lung and liver micro-metastasis nodes in 4T1-bearing mice (**Figure 5C**). Then, we also observed that ALA treatment not only enhanced NRF2 and p-NRF2 levels (**Figure 5D**) but also increased metastasis-associated proteins including MMP-2, MMP-9, and VEGF levels in lung and liver metastasis tissues (**Figure 5E**), indicating that NRF2 activation can promote BC metastasis by increasing metastasis-associated genes. Together, our results strongly suggest that NRF2 activation plays an oncogenic role in DPP-4i–driven BC metastasis.

### Heme Oxygenase 1 (HO-1) Is a Critical Mediator of Oncogenic ROS-NRF2 Axis in DPP-4i–Driven BC Metastasis

**Figure 6:**
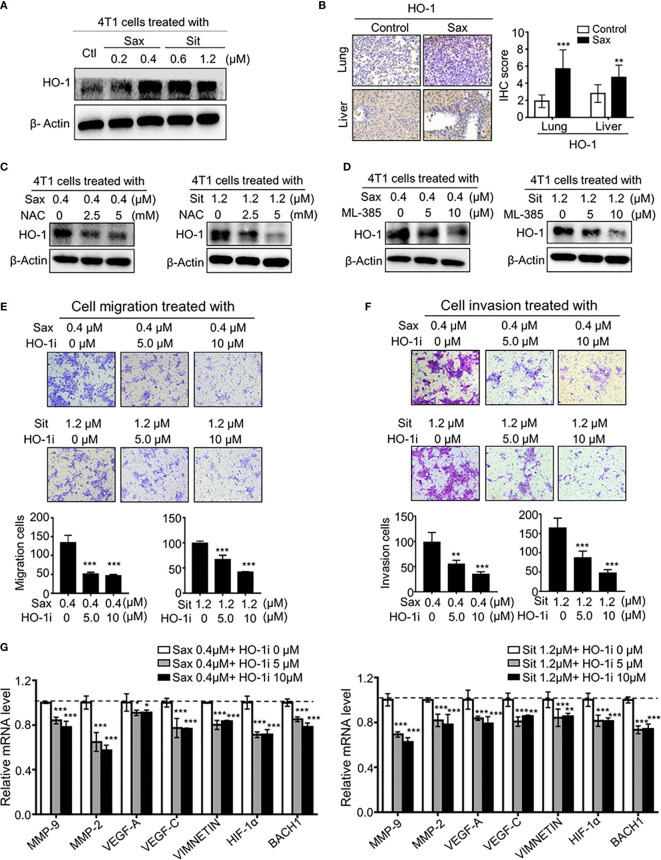
ROS-NRF2 dependent HO-1 activation is critical to DPP-4i-driven BC metastasis. **(A, B)** DPP-4i enhances NRF2-responsive HO-1 activation. HO-1 expression was analyzed by western blotting in DPP-4i-treated 4T1 cells **(A)** and by IHC staining in lung and liver metastasis tissues **(B)**, respectively. **(C)** ROS inhibition abrogates DPP4i-induced HO-1 activation. 4T1 cells were co-treated with DPP-4i and NAC and HO-1 expression were detected by western blotting. β-actin was a loading control. **(D)** NRF2 inhibition attenuates DPP-4i–induced HO-1 activation. 4T1 cells were co-treated with DPP-4i and NRF2 specific inhibitor ML-385, and HO-1 expression were detected by western blotting. β-actin was a loading control. **(E, F)** HO-1 blockage reverses DPP-4i -driven BC cell migration and cell invasion *in vitro*. 4T1 cells were subject to cell migration **(E)** and cell invasion **(F)** upon co-treatment with DPP-4i and HO-1 specific inhibitor for 24 h. Migration or invasion cells were counted in 5 to 10 random fields. **(G)** HO-1 blockage abrogates DPP-4i–driven metastasis-associated gene levels. 4T1 cells were co-treated with or without DPP-4i and HO-1 inhibitor for 4h and metastasis-associated gene levels were detected by qRT-PCR. β-Actin gene was as an internal control. Data are presented as mean ± SD of three independent experiments. Representative images are shown. Scale bar: 50 μm. *p<0.05, **p<0.01, and *** p<0.001 between the indicated groups determined by one-way analysis of variance (ANOVA).

Given the oncogenic role of ROS-NRF2 activations in DPP-4i-driven BC metastasis, we sought to explore the underlying mechanism of how ROS-NRF2 activation promotes BC metastasis. Recent studies reported aberrant HO-1 activation in human cancers (18–22), promoting us to focus on the role of NRF2-responsive HO-1 in DPP-4i-driven BC metastasis. Firstly, we investigated whether DPP-4i could increase HO-1 activation *via* ROS-NRF2 axis-dependent manner. Using Western blot and IHC analysis, we observed that DPP-4i significantly increased HO-1 expression *in vitro* and *in vivo* (**Figures 6A, B**). In addition, we also found that DPP-4i-driven HO-1 levels were significantly abrogated upon NAC treatment or ML-385 intervention (**Figures 6C, D** and **Supplementary Figure S3**). These data indicate that DPP-4i–driven HO-1 activation is ROS-NRF2 axis-dependent in BC cells.

To further define the critical role of HO-1 in DPP-4i–driven BC metastasis, using HO-1-IN-1 hydrochloride, a specific HO-1 inhibitor (12), we found that HO-1 inhibition significantly decreased DPP-4i–driven BC cell migration and invasion (**Figures 6E, F**). Furthermore, we observed that DPP-4i–driven metastasis-associated gene levels were significantly attenuated after HO-1 inhibition in BC cells (**Figure 6G**), suggesting that HO-1 inhibition could reverse DPP-4i–driven BC metastasis. Overall, these results suggest that HO-1 plays a critical role in oncogenic ROS-NRF2 axis-driven BC metastasis.

### HO-1 Promotes BC Metastasis by Activating NRF2 *via* a Positive-Feedback Loop

**Figure 7:**
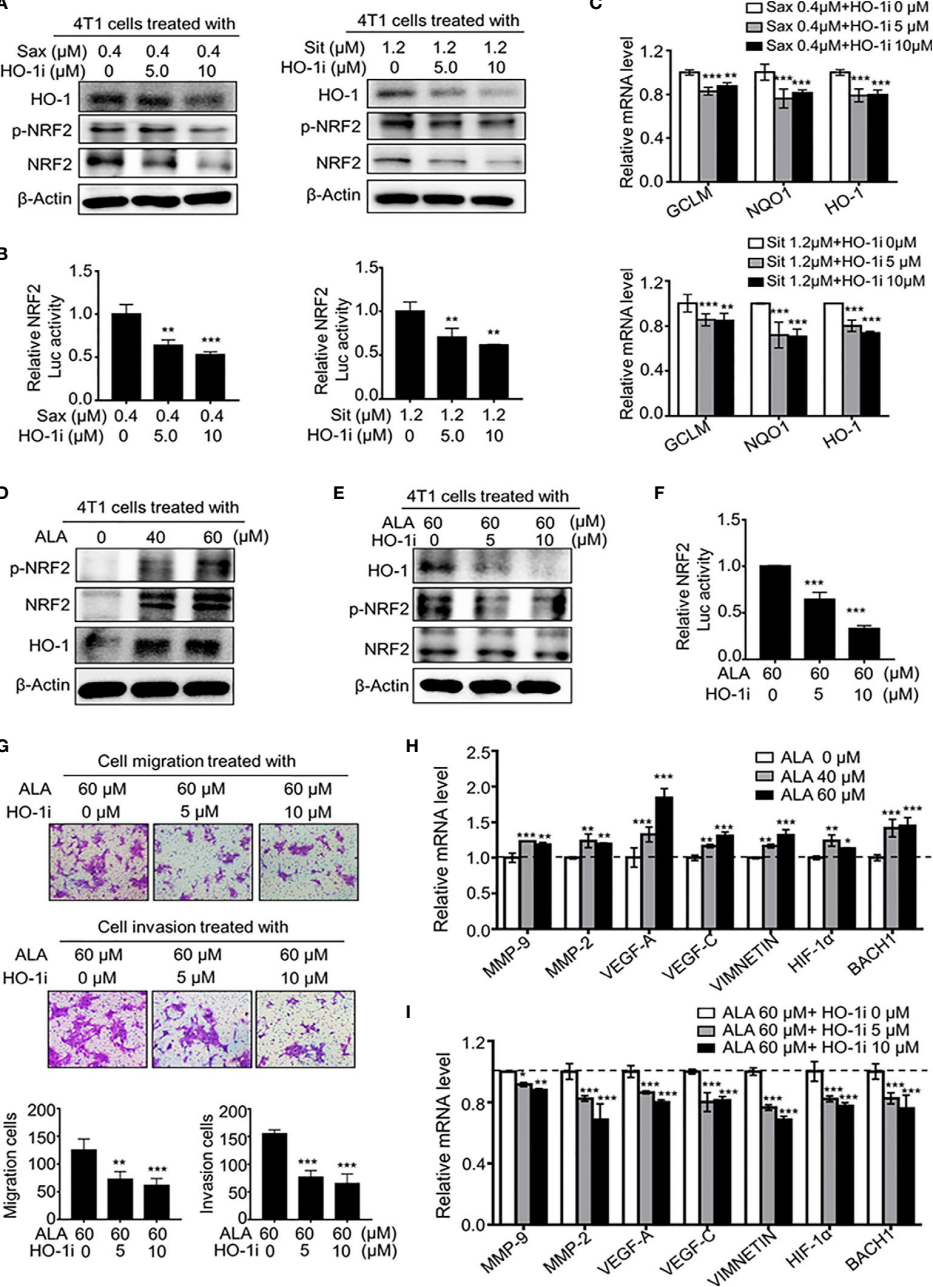
HO-1 reinforces NRF2 activation *via* a positive feedback loop. **(A–C)** HO-1 blockage abrogates DPP-4i-driven NRF2 activation in vitro. 4T1 cells were co-treatment with DPP-4i and HO-1 specific inhibitor (HO-1i). HO-1, p-NRF2, and NRF2 expressions were detected by western blotting **(A)** and NRF2/ARE transcriptional activation was analyzed by luciferase reporter gene assay **(B)**. NRF2-responsive genes were detected by qRT-PCR **(C)**. β-Actin gene was as a control. **(D)** NRF2 activator increases NRF2 activation in 4T1 cells. p-NRF2, NRF2, and HO-1 expressions in ALA-treated 4T1 cells were detected by Western blotting. β-actin was as a loading control. **(E, F)** HO-1 blockage abrogates ALA-induced NRF2 activation. 4T1 cells were co-treated with or without ALA and HO-1 inhibitor. HO-1, p-NRF2 and NRF2 expressions were analyzed by Western blotting **(E)**. NRF2/ARE transcriptional activation was detected by luciferase reporter gene assay **(F)**. **(G)** HO-1 inhibition attenuates ALA-induced BC migration and cell invasion. 4T1 cells were co-treated with ALA and HO-1 inhibitor. Cell migration and cell invasion assays were performed and migration or invasion cells were counted in 5 to 10 random fields. **(H, I)** HO-1 blockage abrogates ALA-driven metastasis-associated gene levels. 4T1 cells were treated with ALA alone **(H)** or were co-treated with ALA and HO-1 inhibitor for 4h **(I)**. Metastasis-associated gene levels were detected by qRT-PCR. β-actin gene was as an internal control. Representative images are shown. Data are presented as mean ± SD of three independent experiments. Scale bar: 50 μm. *p<0.05, **p<0.01, and ***p<0.001 between the indicated groups determined by one-way analysis of variance (ANOVA).

Given the oncogenic role of NRF2 and HO-1 in DPP-4i -driven BC metastasis, we further defined the possible mechanism underlying how NRF2-responsive HO-1 activation promotes BC metastasis. In BC cells, we observed that the HO-1 inhibitor not only decreased HO-1 expression but also significantly attenuated the DPP-4i–driven NRF2 and p-NRF2 expression (**Figure 7A**). Moreover, DPP-4i–driven NRF2/ARE transcriptional activation (**Figure 7B)** and NRF2-responsive genes were also markedly abrogated by HO-1 inhibition (**Figure 7C**), indicating that HO-1 may feedback regulate NRF2 activation in DPP-4i–treated BC cells.

To investigate whether HO-1 inhibition could attenuate NRF2-driven BC metastasis, we conducted a dual pharmacological intervention assay using HO-1 inhibitor and NRF2 activator in BC cells. As shown in **Figures 7D, E**, ALA treatment markedly enhanced NRF2, p-NRF2 and HO-1 expressions, whereas HO-1 inhibition significantly abrogated ALA-induced HO-1, NRF2 and p-NRF2 expressions. Meanwhile, ALA-induced NRF2/ARE transcriptional activation was also attenuated by HO-1 inhibition (**Figure 7F**), indicating a positive-feedback regulation between HO-1 and NRF2. Furthermore, we observed that ALA-driven cell migration and invasion were significantly reversed by HO-1 inhibitor in BC cells (**Figure 7G**), while ALA-induced NRF2 downstream targets as well as metastasis-associated genes levels were markedly inhibited by HO-1 inhibition (**Figures 7H, I** and **Supplementary Figure S2B**), suggesting HO-1 inhibition can directly antagonize NRF2-driven BC metastasis *in vitro*. Thus, these results suggest that HO-1 activation contributes to oncogenic NRF2 activation *via* a positive-feedback loop, promoting a serial of metastasis-associated gene expressions, thereby leading to BC metastasis.

## Discussion

Elucidating the potential side effect of antidiabetic agents in BC metastasis may shed new light on minimizing the risk of DPP-4i in diabetic patients with BC. 4T1 cells originally isolated from BALB/c mice share many characteristics with naturally occurring human breast cancer and metastasize to distant organs *via* the hematogenous route, making it as an ideal model for mimicking the metastatic and advanced stages of human breast cancer (11). In our efforts to understand the effect of antidiabetic agents in existing BC, we utilized severe combined immunodeficient NOD-SCID mice to establish a spontaneous BC metastasis mice model to better understand the effect of DPP-4i on biological behaviors of BC cells itself under immune-deficient status. Here, we revealed that DPP-4i promoted BC metastasis *in vitro* and *in vivo*, which can be abrogated by the pharmacological inhibition of ROS, NRF2 and HO-1 respectively. Mechanistically, DPP-4i induced excessive oxidative stress by triggering ROS production, resulting in oncogenic NRF2 and HO-1 activations. Moreover, HO-1 activation promoted oncogenic NRF2 activation *via* a positive feedback loop, promoting a series of metastasis-associated genes expression. Our study provides a critical profile of DPP-4i and aberrant NRF2 activation in BC metastasis.

Our finding demonstrates that DPP-4i promotes BC metastasis by triggering oxidative stress *via* ROS generation, improving our understanding of the role of oxidative stress in BC progression. Our previous finding suggested that DPP-4i promoted human BC metastasis through NRF2-mediated anti-oxidative stress (6). Here, our data clearly showed DPP-4i promoted BC metastasis by triggering aberrant oxidative stress *via* ROS overproduction while ROS scavenger significantly attenuated DPP-4i–induced BC metastasis *in vitro*. However, recent contradicting results have raised a possibility that ROS limits distant metastasis (6, 18). There is a possibility that specific characteristics of antioxidants and cell lines with different gene mutations may contribute to the contradictory results regarding the role of ROS and antioxidant regents in tumor metastasis (6). Our results strongly suggest that ROS-mediated oxidative stress plays an essential role in DPP-4i–driven BC metastasis, further improving our understanding of the role of DPP-4i in the BC progression.

Our study also reveals an oncogenic NRF2 activation in DPP-4i–induced BC metastasis, improving our understanding of the side-effect of DPP-4i in tumor progression. NRF2 has long been identified as a well-known anti-cancer molecular while a serial of antioxidants suppresses tumor progression (19, 20). However, recent evidences suggest the oncogenic profile of NRF2 activation in tumor progression and show that aberrant NRF2 accelerates cancer progression while antioxidants accelerate migration and invasion of cancer cells (6, 21, 22). Our recent report also showed that mitochondrial GRIM-19 deficiency accelerated human gastric cancer metastasis through the oncogenic ROS-NRF2-HO-1 axis *via* a positive-feedback NRF2-HO-1 loop. Therefore, this study not only offers novel insights to the oncogenic role of NRF2 in BC progression, but also provides new strategies to alleviate the dark side of NRF2 by targeting HO-1. Here, our data demonstrated that DPP-4i promoted BC metastasis by ROS-dependent NRF2 activation while specific inhibition of ROS or NRF2 abrogated DPP-4i-driven BC metastasis, which is consistent with recent findings that NRF2 inhibitors could antagonize human cancers (23–25). Therefore, our results strongly suggest that NRF2 plays an oncogenic role in DPP-4i–driven BC metastasis.

Our finding reveals that NRF2-responsive HO-1 contributes to DPP-4i–induced oncogenic NRF2 activation *via* a positive-feedback NRF2-HO-1 loop, not only providing novel mechanism insights into the oncogenic NRF2 in BC metastasis, but also offering new strategies to inhibit the dark side of oncogenic NRF2 activation by targeting its downstream target HO-1. Our results demonstrate that NAC suppresses DPP-4i-driven BC metastasis whereas NRF2 activator ALA accelerates BC metastasis, raising a topic concerning the paradox profile of antioxidants in cancer progression. HO-1, as one of NRF2 downstream targets, plays a critical role in the maintenance of cellular redox homeostasis (12, 21). However, recent evidences also showed that aberrant HO-1 in human cancers contributes to cancer metastasis (12, 26–30). Here, our results showed that the pharmacological ROS or NRF2 inhibition decreased HO-1 level while HO-1 inhibition abrogated DPP-4i-driven BC metastasis, suggesting that NRF2-responsive HO-1 activation is required to DPP-4i-driven BC metastasis. Moreover, recent finding also showed that HO-1 activation was correlated with metastasis-associated genes such as MMP-9, VEGF-A, HIF-1α, and BACH-1 (26–28, 31–33). Our data also showed that HO-1 inhibition blocked DPP-4i-driven metastasis-associated genes in BC cells. Our recent report also showed that oncogenic ROS-NRF2-HO-1 axis contributes to mitochondrial GRIM-19 deficiency-driven metastasis in human gastric cancer *via* a positive-feedback NRF2-HO-1 loop (12). Thus, HO-1, as a critical downstream target of the ROS-NRF2 axis, feedback promotes oncogenic NRF2 activation, resulting in upregulation of metastasis-associated genes, thereby facilitating BC metastasis. Our finding provides a reasonable explanation for the NRF2-driven BC metastasis, and suggests that more comprehensive preclinical and clinical studies should be performed to ensure the safety of antioxidants in cancer patients.

In summary, our data suggest that DPP-4i accelerates murine BC metastasis through the oncogenic ROS-NRF2-HO-1 axis *via* a positive-feedback NRF2-HO-1 loop. This finding not only offers a mechanistic insight to DPP-4i-driven BC metastasis by ROS-NRF2 activation, but also provides new avenues to eliminate the “dark side” of NRF2 by targeting HO-1.

## Data Availability Statement

The raw data supporting the conclusions of this article will be made available by the authors, without undue reservation.

## Ethics Statement

The animal study was reviewed and approved by the Institutional Animal Care and Use Committee of Children’s Hospital of Chongqing Medical University.

## Author Contributions

Conception and design: RL, XZ, MY, YH. Development of methodology: RL, XZ, XW, BX, XX. Acquisition of data (provided animals, provided facilities, etc.): RL, XZ, XW, BX, XX. Analysis and interpretation of data (e.g., statistical analysis): RL, XZ, JF, BX, YH. Writing, review, and/or revision of the manuscript: RL, XZ, MY, LB, YH. Administrative, technical, or material support (i.e., reporting or organizing data, constructing databases): RL, XX, JF, XW, BX. Study supervision: YH. All authors contributed to the article and approved the submitted version.

## Funding

This study was partly supported by Chongqing basic and frontier research project (CSTC2018jcyjAX0218) and Chongqing Yuzhong District Sci & Tech Research Project (20190106).

## Conflict of Interest

The authors declare that the research was conducted in the absence of any commercial or financial relationships that could be construed as a potential conflict of interest.

## Supplementary Material

Supplementary Figure 1 DPP-4i induces aberrant oxidative stress in BC cells. Oxidative stress status in DPP-4i-treated BC cells was evaluated by analyzing ATP content **(A)**, NADP+/NADPH ratio **(B)** and GSH/GSSG ratio **(C)**. Data are presented as mean ± SD of three independent experiments. *p<0.05, **p<0.01, and ***p<0.001 between the indicated groups determined by one-way analysis of variance (ANOVA).

Supplementary Figure 2 HO-1 blockage abrogates ALA-induced NRF2-responsive genes expression. 4T1 cells were treated with ALA **(A)** or were co-treated with ALA and HO-1 inhibitor **(B)**. The expression of NRF2-responsive genes was analyzed by Real-time PCR. β-actin was as an internal control. Data are presented as mean ± SD of three independent experiments. *p<0.05, **p<0.01, and ***p<0.001 between the indicated groups determined by one-way analysis of variance (ANOVA).

Supplementary Figure 3 Relative expression analysis of HO-1 protein after co-treatment of DPP-4i with NAC or ML-385. 4T1 cells were co-treated with DPP-4i and NAC **(A)** or ML-385 **(B)**. HO-1 expression was detected by western blotting as shown in Figure 6C and 6D. Relative HO-1 expression was obtained by analyzing the ratio of HO-1 and β-Actin. Data are presented as mean ± SD of three independent experiments. ** p<0.01 and *** p<0.001 between the indicated groups determined by one-way analysis of variance (ANOVA).
