# Supplementary figures and images for: Antidiabetic Agent DPP-4i Facilitates Murine Breast Cancer Metastasis by Oncogenic ROS-NRF2-HO-1 Axis via a Positive NRF2-HO-1 Feedback Loop
Source: Front Oncol. 2021 May 26;11:679816. doi: 10.3389/fonc.2021.679816 (PMC8187865; doi:10.3389/fonc.2021.679816)

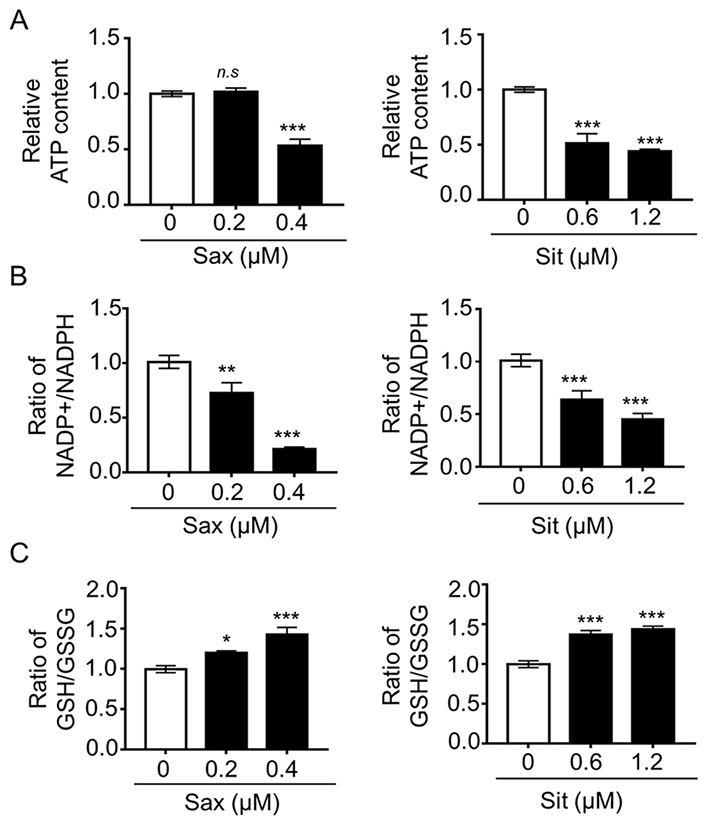

Supplement: Supplementary Figure 1 — DPP-4i induces aberrant oxidative stress in BC cells. Oxidative stress status in DPP-4i-treated BC cells was evaluated by analyzing ATP content (A), NADP+/NADPH ratio (B) and GSH/GSSG ratio (C). Data are presented as mean ± SD of three independent experiments. *p<0.05, **p<0.01, and ***p<0.001 between the indicated groups determined by one-way analysis of variance (ANOVA). [file Image_1.tif]

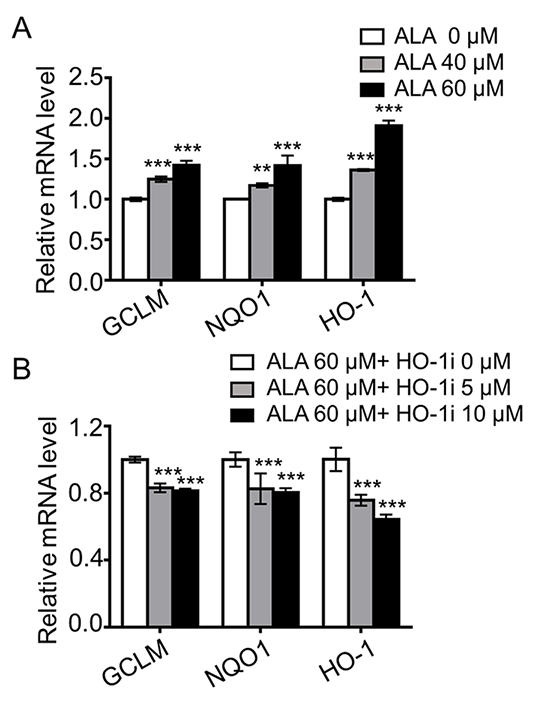

Supplement: Supplementary Figure 2 — HO-1 blockage abrogates ALA-induced NRF2-responsive genes expression. 4T1 cells were treated with ALA (A) or were co-treated with ALA and HO-1 inhibitor (B). The expression of NRF2-responsive genes was analyzed by Real-time PCR. β-actin was as an internal control. Data are presented as mean ± SD of three independent experiments. *p<0.05, **p<0.01, and ***p<0.001 between the indicated groups determined by one-way analysis of variance (ANOVA). [file Image_2.tif]

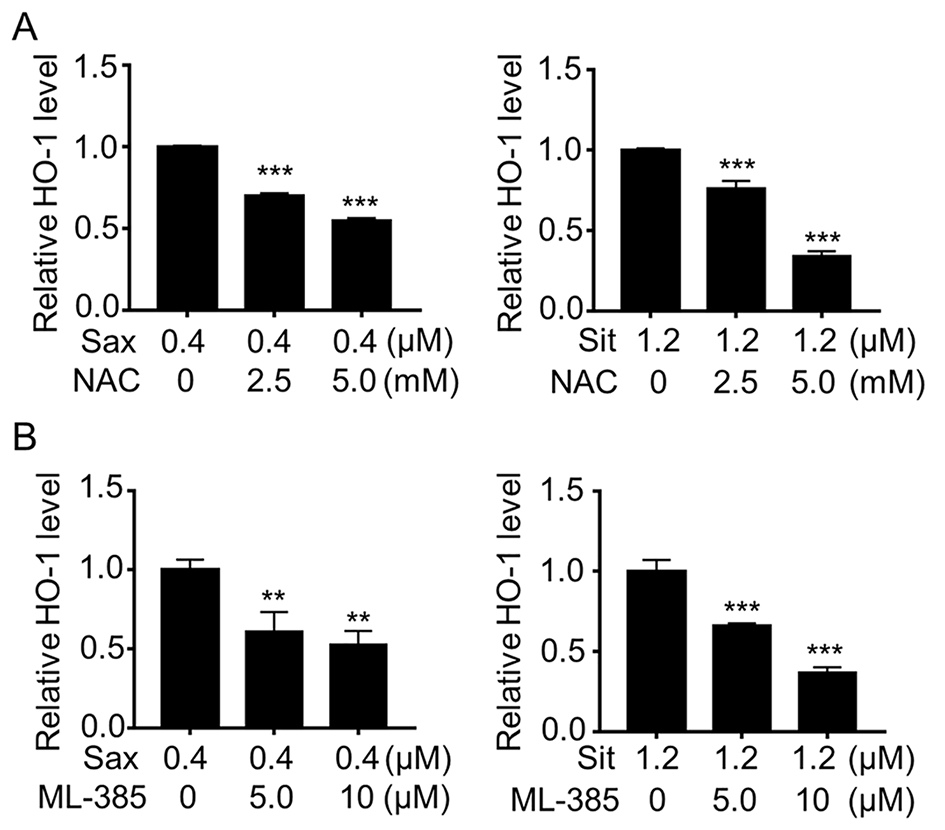

Supplement: Supplementary Figure 3 — Relative expression analysis of HO-1 protein after co-treatment of DPP-4i with NAC or ML-385. 4T1 cells were co-treated with DPP-4i and NAC (A) or ML-385 (B). HO-1 expression was detected by western blotting as shown in Figure 6C and 6D. Relative HO-1 expression was obtained by analyzing the ratio of HO-1 and β-Actin. Data are presented as mean ± SD of three independent experiments. ** p<0.01 and *** p<0.001 between the indicated groups determined by one-way analysis of variance (ANOVA). [file Image_3.tif]
