# Supplementary material for: Antidiabetic Agent DPP-4i Facilitates Murine Breast Cancer Metastasis by Oncogenic ROS-NRF2-HO-1 Axis via a Positive NRF2-HO-1 Feedback Loop
Source: Front Oncol. 2021 May 26;11:679816. doi: 10.3389/fonc.2021.679816 (PMC8187865; doi:10.3389/fonc.2021.679816)
Supplement: Supplementary file 4 [file Table_1.doc]

**Table S1** Mouse primers for Real-time PCR in this study

| Gene (GenBank  Accession No) | Primer sequences  (5'-3') | Annealing  Temp (℃) | | Size  (bp) | |
| --- | --- | --- | --- | --- | --- |
| NRF2  (NM_010902.4) | F: GCCCAGCACATCCAGACAG  R: TATCCAGGGCAAGCGACTCA | | 60 | | 154 |
| NQO1  (NM_008706.5) | F: ATGAAGGAGGCTGCTGTAGAG  R: ATATGCTAGAGATGACTCGGAAGG | | 60 | | 165 |
| GCLM  (NM_008129.4) | F: CACAATGACCCGAAAGAACTG  R: GTAGCCTTTAGACTTGATGATTCC | | 60 | | 75 |
| HO-1  (NM_010442.2)  BACH1  (NM_178309.2)  HIF-1α  (NM_001313919.1)  MMP-9  (NM_013599.4)  MMP-2  (NM_008610.3)  VEGF-A  （NM_001025250.3）  VEGF-C  (NM_009506.2)  VIMENTIN  (NM_011701.4)  β-ACTIN  (NM_007393) | F: CACAAAGACCAGAGTCCCTCA  R: GCAGTATCTTGCACCAGGCT  F: CTCAGGGGTCCCGATGACTA  R: ATAGCAGGACTTCCCGTGC  F: CCTTAACCTGTCTGCCACTTTG  R: TAATGTTCCAATTCCTGCTGCTTG  F: GCCGACTTTTGTGGTCTTCC  R: GGTACAAGTATGCCTCTGCCA  F: CTGCAGGGTGGTGGTCATA  R: TGCTTCACATCCTTCACCTGG  F: GCAGAAGTCCCATGAAGTGAT  R: CACTCCAGGGCTTCATCGTT  F: GGCGAGGTCAAGGCTTTT  R: TGGGTACAGGACAGACATCA  F: TGGTACAAGTCCAAGTTTGCT  R: TCTCCGGTACTCGTTTGACT  F: GGCTGTATTCCCCTCCATCG  R: CCAGTTGGTAACAATGCCATGT | | 60  60  60  60  60  60  60  60  60 | | 71  130  148  80  182  160  90  96  154 |

F: Forward; R: Reverse;
