# Supplementary material for: Antidiabetic Agent DPP-4i Facilitates Murine Breast Cancer Metastasis by Oncogenic ROS-NRF2-HO-1 Axis via a Positive NRF2-HO-1 Feedback Loop
Source: Front Oncol. 2021 May 26;11:679816. doi: 10.3389/fonc.2021.679816 (PMC8187865; doi:10.3389/fonc.2021.679816)
Supplement: Supplementary file 5 [file Table_2.doc]

**Table S2** Antibodies information used in this study

| Antibodies | Catalog Number | | Dilution  for IHC | Dilution  for IF | Dilution  for WB |
| --- | --- | --- | --- | --- | --- |
| NRF2 | Bioss Inc | bs-1074R | 1:500 | / | 1:500 |
| P-NRF2 | Bioss Inc | bs-2013R | 1:500 | / | 1:500 |
| HO-1 | Santa Cruz | sc-390991 | 1:300 | / | 1:300 |
| 8-OHdG | Bioss Inc | bs-1278R | / | 1:200 | / |
| β-actin | Sigma | A5441 | / | / | 1:7000 |
| VEGF | Bioss Inc | bs-0279R | 1:400 | / | / |
| MMP-2 | Bioss Inc | bs-0412R | 1:400 | / | / |
| MMP-9 | Bioss Inc | bs-4593R | 1:400 | / | / |
| Vimentin | Bioss Inc | bs-0756R | 1:400 | / | / |

Note: Bioss Inc: Beijing Biosynthesis Biotechnology Co., Ltd.
